# Detectable chromosome X mosaicism in males is rarely tolerated in peripheral leukocytes

**DOI:** 10.1038/s41598-020-80948-0

**Published:** 2021-01-13

**Authors:** Weiyin Zhou, Shu-Hong Lin, Sairah M. Khan, Meredith Yeager, Stephen J. Chanock, Mitchell J. Machiela

**Affiliations:** 1 Division of Cancer Epidemiology and Genetics, National Cancer Institute, 9609 Medical Center Drive, Rockville, MD 20850 USA; 2 Cancer Genomics Research Laboratory, Frederick National Laboratory for Cancer Research, Rockville, MD 20850 USA

**Keywords:** Computational biology and bioinformatics, Genetics

## Abstract

Age-related male Y and female X chromosome mosaicism is commonly observed in large population-based studies. To investigate the frequency of male X chromosome mosaicism, we scanned for deviations in chromosome X genotyping array intensity data in a population-based survey of 196,219 UK Biobank men. We detected 12 (0.006%) men with mosaic chromosome X gains ≥ 2 Mb and found no evidence for mosaic chromosome X loss, a level of detection substantially lower than for autosomes or other sex chromosomes. The rarity of chromosome X mosaicism in males relative to females reflects the importance of chromosome X gene dosage for leukocyte function.

## Introduction

Genetic mosaicism is the presence of clonal populations of cells harboring post-zygotic mutations. The size of mosaic mutations ranges from single point mutations to large somatic copy number (or neutral) alterations (SCNAs) spanning an entire chromosome^1–7^. Genetic mosaicism is observed in normal tissue, with increasing levels detected as the sensitivity of detection approaches improves^8–10^. Recent evidence suggests SCNAs may be associated with elevated risk for chronic diseases like cancer^11–14^. Population-based studies have characterized age-related mosaic SCNAs in DNA of circulating leukocytes from apparently healthy individuals using genotyping array-based detection approaches^3–7^. Autosomal mosaicism is observed in approximately 5% of adults^6,7^, mosaic Y loss (mLOY) is estimated to affect over 15% of elderly men^11–13^, and female X chromosome mosaicism is observed at 4 times the rate of the autosomes^15^. Reports of female X chromosome mosaicism indicate events more often involve the entire chromosome and favor the inactivated X chromosome. This is likely due to the ability of female leukocytes to tolerate loss or gain of a Barr body^15^. Female tumor genomes also have elevated rates of mutation (2–4 × per Mb) on the inactivated X chromosome^16^.

The frequency of male X chromosome mosaicism has not been well-characterized in large populations. In females, who are diploid for the X chromosome, the loss or gain of an inactivated copy of the X chromosome can be tolerated. In hemizygous males, changes in X chromosome dosage, particularly losses, may be deleterious for cellular function and survival.

## Results

**Figure 1:**
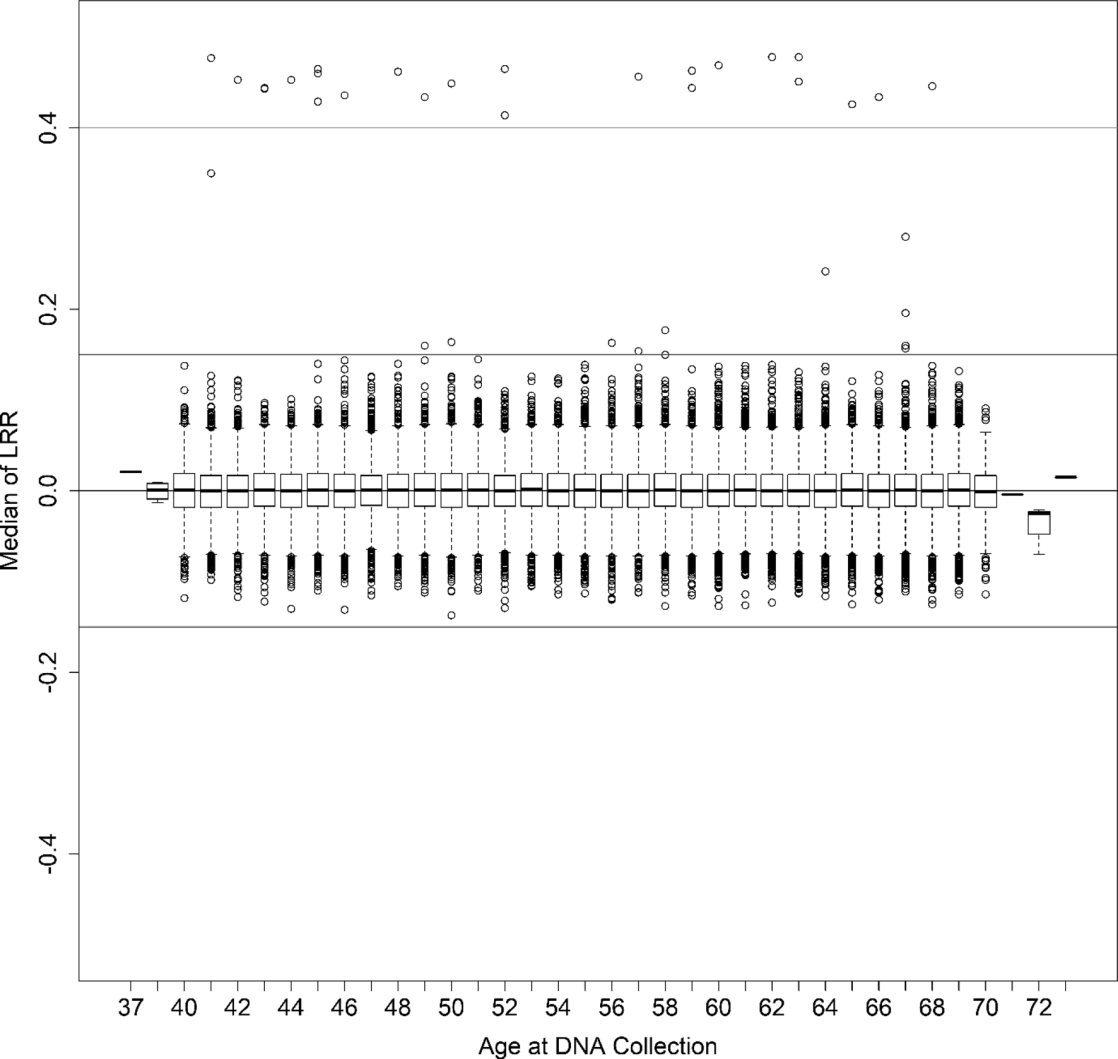
Boxplot of baseline corrected median chromosome X log_2_ R ratio (LRR) at age of sample collection for 196,219 male UK Biobank participants. Distribution of baseline corrected median LRR is plotted by 1-year age groups. Male baseline corrected LRR values are centered around 0. Instances where baseline corrected median LRR ≥ |0.15| (blue lines) indicate evidence for chromosome X mosaicism. Participants with LRR ≥ 0.4 (green line) are constitutional XXY (Klinefelter syndrome).

We detected a lower than expected total of 12 (0.006%) mosaic gains, including one on the q arm of the X chromosome (Xq) and 11 spanning the entire X chromosome (Fig. 1, Supplementary Fig. S1). Most detected gains were at low cellular fractions, Log_2_ R Ratio (LRR)-estimated proportions of 11–18% (Supplementary Table S1). Only two detected mosaic chromosome X gains had median LRR ≥ 0.25, suggesting that it is rare to observe ≥ 20% of leukocytes affected by mosaic X chromosome gains (Supplementary Table S1). B allele frequency (BAF) plots indicated no heterozygosity apart from pseudoautosomal regions and the X-transposed region (Xq21) (Supplementary Figure S1), suggesting mitotic nondisjunction as the most likely origin; although revertant constitutional XXY isodisomy could be possible. We did not detect evidence for mosaic chromosome X loss in any men. We detected 24 (0.01%) men with constitutional XXY (Supplementary Table S2, Supplementary Figs. S2–S3), lower than the estimated 0.15% in the UK population^17^ likely due to the highly selected and generally healthy population participating in the UK Biobank^18^. We observed 3 cases with heterozygosity spanning the entire X chromosome suggesting paternal meiosis I errors or maternal meiosis I errors in the absence of recombination as the molecular origin of heterodisomy. An additional 10 cases of detected constitutional XXY had partial heterozygous BAF plots suggesting nondisjunction errors during maternal meiosis I or II when recombination previously occurred resulting in partial heterodisomy. The remaining 11 cases demonstrated no evidence for X chromosome heterozygosity indicating isodisomy because of female meiotic II errors in the absence of recombination or early post-zygotic mitotic errors. All XXY men had normal Y chromosome dosage, except for one individual (K2) who displayed LRR evidence for a Y chromosome gain (Supplementary Fig. S3).

## Discussion

The rarity of X mosaicism in UK Biobank males (0.006%) likely reflects the deleterious impact on cell survival or clonal fitness of losing key cellular genes on a hemizygous chromosome. It is unlikely that the lack of evidence for detectable male chromosome X mosaicism, particularly loss of the entire chromosome, is primarily due to the analytical approach. We note that detection of mosaic events on the male X chromosome from array intensity data requires a specialized detection approach. Autosomal and female X chromosome mosaic events are detected from array intensity data using either segmentation of BAF data of heterozygous variants^3–6^ or a combination of BAF and genotype phase data^7,19^. The male X chromosome is hemizygous and does not have BAF values for heterozygous variants. Instead, variant LRR data is used for investigating sex chromosome (X and Y chromosome) mosaicism in men, as exemplified by several studies of mosaic chromosome Y loss^11–14,20^. Our detection of male chromosome X mosaicism is based on LRR data, with an additional step required for correction of the proportion of males and females present in the UK Biobank genotyping cluster files. Our LRR detection approach effectively centers median chromosome X LRR values around the expected value of zero and suggests that if males harbor X chromosome mosaicism, the fraction of abnormal cells is low and below the limit of detection of existing genotyping array-based approaches (i.e., < 10% cellular fraction). It is plausible that male X mosaicism is present in circulating leukocytes below levels of current detection, but this would involve a low fraction of circulating cells suggesting a challenge to leukocyte fitness.

The disparity between high rates of chromosome Y mosaicism, specifically mLOY, and the relative rarity of X mosaicism in men likely reflects the biological importance of gene function and dosage on these sex chromosomes. The X chromosome contains more than 1000 genes, many of which are established as essential in cellular function^21,22^, while the male specific region of the Y chromosome only encodes 27 proteins^23^. The lack of evidence for clonal fitness for mosaic loss of X chromosomes in men is striking and underscores the importance of single copy genes on the X chromosome. This is consistent with X chromosome evolutionary history in humans and other mammals, wherein purifying (negative) selection has accelerated the removal of deleterious alleles on the X chromosome relative to the autosomes^24^. Genomic investigations characterizing tumor DNA also demonstrate an absence of X nullisomy in cancer genomes; although chromosome Y loss is common^25–27^. The Y chromosome contains substantially fewer genes, many of which have roles in sex-determination and spermatogenesis^21–23^, but their loss can be tolerated with aging. Evidence from our large population-based investigation of men suggests chromosome X mosaicism is rare in blood; therefore, making it unlikely that male X mosaicism could have large attributable fractions for risk of common cancers or chronic diseases. Our findings underscore the importance of gene dosage in sex chromosomes, particularly in men.

## Conclusions

Our analysis identified low frequencies of X chromosome mosaicism in circulating leukocytes of men. While cases of Klinefelter syndrome demonstrate that male cells can tolerate extra copies of the X chromosome, albeit with known phenotypic consequences such as elevated breast cancer rates^28–30^, our findings suggest clonal expansion of leukocytes harboring a change in X chromosome dosage is rare in the general male population and, when present, is a mosaic gain in usually less than 20% of leukocytes. These observational findings in UK Biobank men are in agreement with the biological hypothesis that X inactivation ensures proper X chromosome dosage in females as well as in cases of Klinefelter syndrome for normal cellular function^31,32^.

## Materials and methods

We characterized the frequency of chromosome X mosaicism in males scanning for large-scale chromosome X mosaicism (≥ 2 Mb) in previously genotyped DNA samples from 196,219 male UK Biobank participants. The UK Biobank is a population-based cohort study representing individuals in the UK's National Health Service, who reside near a UK Biobank assessment center. UK Biobank participants provided baseline information on demographic, lifestyle, and other health-related factors, as well as biological samples and physical measures. The National Information Governance Board for Health and Social Care and the National Health Service North West Multicentre Research Ethics Committee approved the UK Biobank study and all participants provided written informed consent at enrollment. The study was conducted in accordance with recognized ethical guidelines (e.g., U.S. Common Rule). DNA samples were blood-derived with age at sample collection ranging from 37 to 73 years of age (mean = 56.76, median = 58). Male UK Biobank participants were previously genotyped using either Affymetrix UK BiLEVE or UK Biobank Axiom arrays^18,33^. All genomic and covariate data used in this publication is available through application for access through the UK Biobank (https://www.ukbiobank.ac.uk/register-apply/).

Log_2_ R Ratio (LRR) was used to detect possible mosaic gains and losses of the male X chromosome. LRR values are the log_2_ of the ratio of observed SNP intensity value to expected intensity value. Deviations in LRR values greater than expected baseline LRR suggest copy number gain and less than expected baseline LRR suggest copy number loss. We used two median LRR thresholds for detecting potential mosaic events that reflect different leukocyte proportions affected by X mosaicism: moderate: |LRR| ≥ 0.15 and high: |LRR| ≥ 0.25. As the X chromosome in males is hemizygous, it was not possible to investigate potential copy neutral mosaic events. We calculated median and standard deviation of LRR across all probes on the X chromosome and filtered out samples with high variability in LRR values (standard deviation of LRR ≥ 0.30). Our final analytic sample size consisted of 196,219 men.

As raw UK Biobank LRR values for males were calculated by combining intensity data together for both males and females (Supplementary Fig. S4), detection of male X mosaicism required generation of a male-specific adjusted LRR so baselines for males would be centered around 0 (Fig. 1) for correct copy number calling. We calculated male-specific LRR values for each genotyping probe on the X chromosome by taking the median of LRR values for each probe across all male samples. A baseline-corrected LRR was then generated by subtracting this male- specific baseline LRR from each male sample LRR in the genotyping cluster group. The male X chromosome baseline-corrected LRR values for each male participant were then segmented for mosaic events using the circular binary segmentation (CBS) algorithm in the R package, DNAcopy^34^ by applying the following workflow: (1) perform outlier detection and smoothing of the LRR data prior to analysis by circular binary segmentation, (2) segment baseline corrected LRR data into regions of estimated equal copy number using circular binary segmentation (CBS), (3) run the analysis with and without the “undo splits” option to remove change-points that are likely due to local trends (< 3 standard deviations apart), (4) filter to select for events with median LRR ≥ 0.15 and ≥ 0.25 and the number of probes ≥ 200 for possible gain as well as median of segmentation region ≤  − 0.15 and ≤  − 0.25 and the number of probes ≥ 200 for possible loss, (5) plot all detected potential gains and losses for manual review by a trained reviewer, and (6) check chromosome Y probes for all cases of potential gain of the X chromosome to ensure each case is karyotypically male.

## Supplementary Information

Supplementary Information.

## Data Availability

All genomic and covariate data used in this publication is available through application for access through the UK Biobank (https://www.ukbiobank.ac.uk/register-apply/).
