# Supplementary material for: Detectable chromosome X mosaicism in males is rarely tolerated in peripheral leukocytes
Source: Sci Rep. 2021 Jan 13;11:1193. doi: 10.1038/s41598-020-80948-0 (PMC7806852; doi:10.1038/s41598-020-80948-0)

**Supplementary Materials**

**Detectable chromosome X mosaicism in males is rarely tolerated in peripheral leukocytes**

Weiyin Zhou, Shu-Hong Lin, Sairah M. Khan, Meredith Yeager, Stephen J. Chanock and Mitchell J. Machiela

**Supplementary Tables**

**Supplementary Table S1.**

| **Subject** | **Chromosome X Start Coordinate** | **Chromosome X End Coordinate** | **Number of Probes Spanning Event** | **Event LRR**  **Median** | **LRR Estimated Cellular Proportion** | **Called Copy Number Status** | **Age (Years)** |
| --- | --- | --- | --- | --- | --- | --- | --- |
| M1 | 132161755 | 152930382 | 3251 | 0.196 | 0.15 | Gain (q arm) | 67 |
| M2 | 2699676 | 154923374 | 18857 | 0.150 | 0.11 | Gain | 58 |
| M3 | 2699676 | 154923374 | 18857 | 0.154 | 0.11 | Gain | 57 |
| M4 | 2699676 | 154923374 | 18857 | 0.157 | 0.12 | Gain | 67 |
| M5 | 2699676 | 154923374 | 18857 | 0.160 | 0.12 | Gain | 49 |
| M6 | 2699676 | 154923374 | 18857 | 0.160 | 0.12 | Gain | 67 |
| M7 | 2699676 | 154923374 | 18857 | 0.163 | 0.12 | Gain | 56 |
| M8 | 2699676 | 154923374 | 18857 | 0.164 | 0.12 | Gain | 50 |
| M9 | 2699676 | 154923374 | 18857 | 0.177 | 0.13 | Gain | 58 |
| M10 | 2699676 | 154923374 | 18857 | 0.242 | 0.18 | Gain | 64 |
| M11 | 2699676 | 154923374 | 18857 | 0.280 | 0.22 | Gain | 67 |
| M12 | 2699676 | 154923374 | 18857 | 0.350 | 0.28 | Gain | 41 |

Characteristics of array-detected male chromosome X mosaic events in the UK Biobank male population. Events for each subject are displayed with chromosome location (GRChb37), number of probes covered and Log_2_ R Ratio (LRR) metrics for the detected event. Copy number status indicates all events were mosaic gains. Age at sample collection is included for reference.

**Supplementary Table S2.**

| **Subject** | **X Chromosome Status** | **Event LRR Median** | **Y Chromosome Status** | **Genotype** | **Age (Years)** |
| --- | --- | --- | --- | --- | --- |
| K1 | XX (unique) | 0.477 | Y | XXY | 41 |
| K2 | XX (unique) | 0.453 | Y gain | XXYY | 42 |
| K3 | XX (UPD) | 0.443 | Y | XXY | 43 |
| K4 | XX (UPD) | 0.444 | Y | XXY | 43 |
| K5 | XX (Partial UPD) | 0.453 | Y | XXY | 44 |
| K6 | XX (unique) | 0.429 | Y | XXY | 45 |
| K7 | XX (Partial UPD) | 0.465 | Y | XXY | 45 |
| K8 | XX (Partial UPD) | 0.460 | Y | XXY | 45 |
| K9 | XX (Partial UPD) | 0.436 | Y | XXY | 46 |
| K10* | XX (Partial UPD) | 0.462 | Y | XXY | 48 |
| K11 | XX (UPD) | 0.434 | Y | XXY | 49 |
| K12 | XX (Partial UPD) | 0.449 | Y | XXY | 50 |
| K13 | XX (UPD) | 0.414 | Y | XXY | 52 |
| K14* | XX (UPD) | 0.465 | Y | XXY | 52 |
| K15* | XX (UPD) | 0.457 | Y | XXY | 57 |
| K16 | XX (UPD) | 0.463 | Y | XXY | 59 |
| K17* | XX (UPD) | 0.444 | Y | XXY | 59 |
| K18 | XX (Partial UPD) | 0.469 | Y | XXY | 60 |
| K19 | XX (Partial UPD) | 0.478 | Y | XXY | 62 |
| K20 | XX (UPD) | 0.478 | Y | XXY | 63 |
| K21* | XX (UPD) | 0.451 | Y | XXY | 63 |
| K22 | XX (UPD) | 0.426 | Y | XXY | 65 |
| K23 | XX (Partial UPD) | 0.434 | Y | XXY | 66 |
| K24 | XX (Partial UPD) | 0.446 | Y | XXY | 68 |

Inferred X and Y chromosome presence based on B Allele Frequency (BAF) and Log_2_ R Ratio (LRR) plots in **Supplementary Figures 2**-**3** for 24 UK Biobank participants with detected constitutional Klinefelter syndrome. An asterisk (*) indicates the individual was classified as XXY by UK Biobank. X chromosome status of “XX (unique)” implies the presence of two entirely unique X chromosomes. “XX (UPD)” indicates an individual has uniparental disomy of the same identical X chromosome. “XX (Partial UPD)” implies the presence of at least one partial stretch of UPD observed in the BAF plot. A Y Chromosome Status of “Y” indicates the presence of a full Y chromosome (see **Supplementary Figure 3**). “Y gain” suggests evidence for an extra copy of the Y chromosome.

**Supplementary Figure S1.** Baseline corrected Log_2_ R Ratio (LRR) and B Allele Frequency (BAF) plots for all detected chromosome X events from UK Biobank. (**A**) Detected mosaic gain on the q arm of chromosome X (Xq). (**B**) All detected cases of mosaic whole chromosome X gain. The green lines represent LRR = ± 0.15. The blue line represents the median across the X chromosome. The figure continues onto the next page.

**
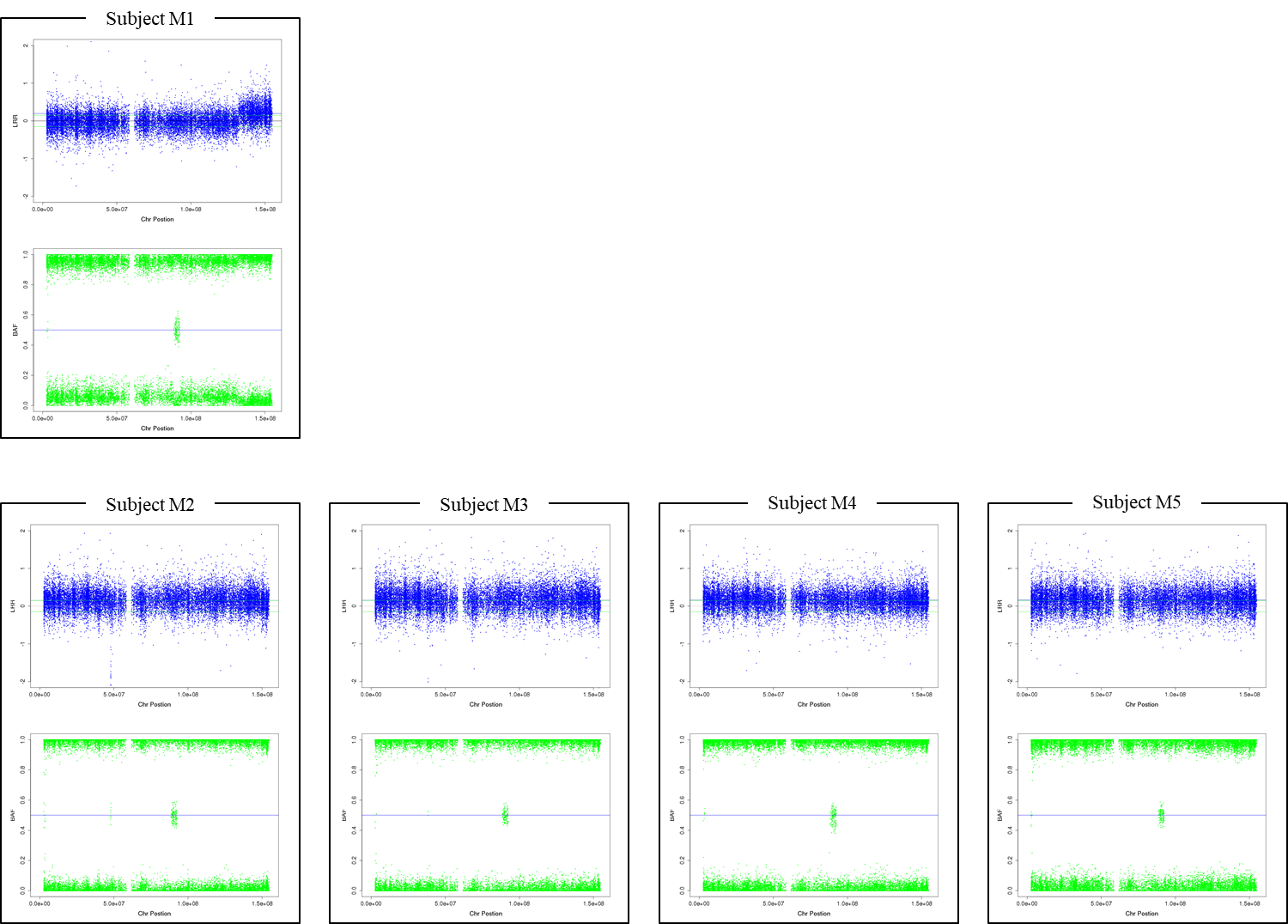
**

**(A)**

**(B)**

**Supplementary Figure S1 (continued)**. Baseline corrected Log_2_ R Ratio LRR and B Allele Frequency (BAF) plots for all detected chromosome X events from UK Biobank. (**A**) Detected mosaic gain on the q arm of chromosome X (Xq). (**B**) All detected cases of mosaic whole chromosome X gain. The green lines represent LRR = ± 0.15. The blue line represents the median across the X chromosome.


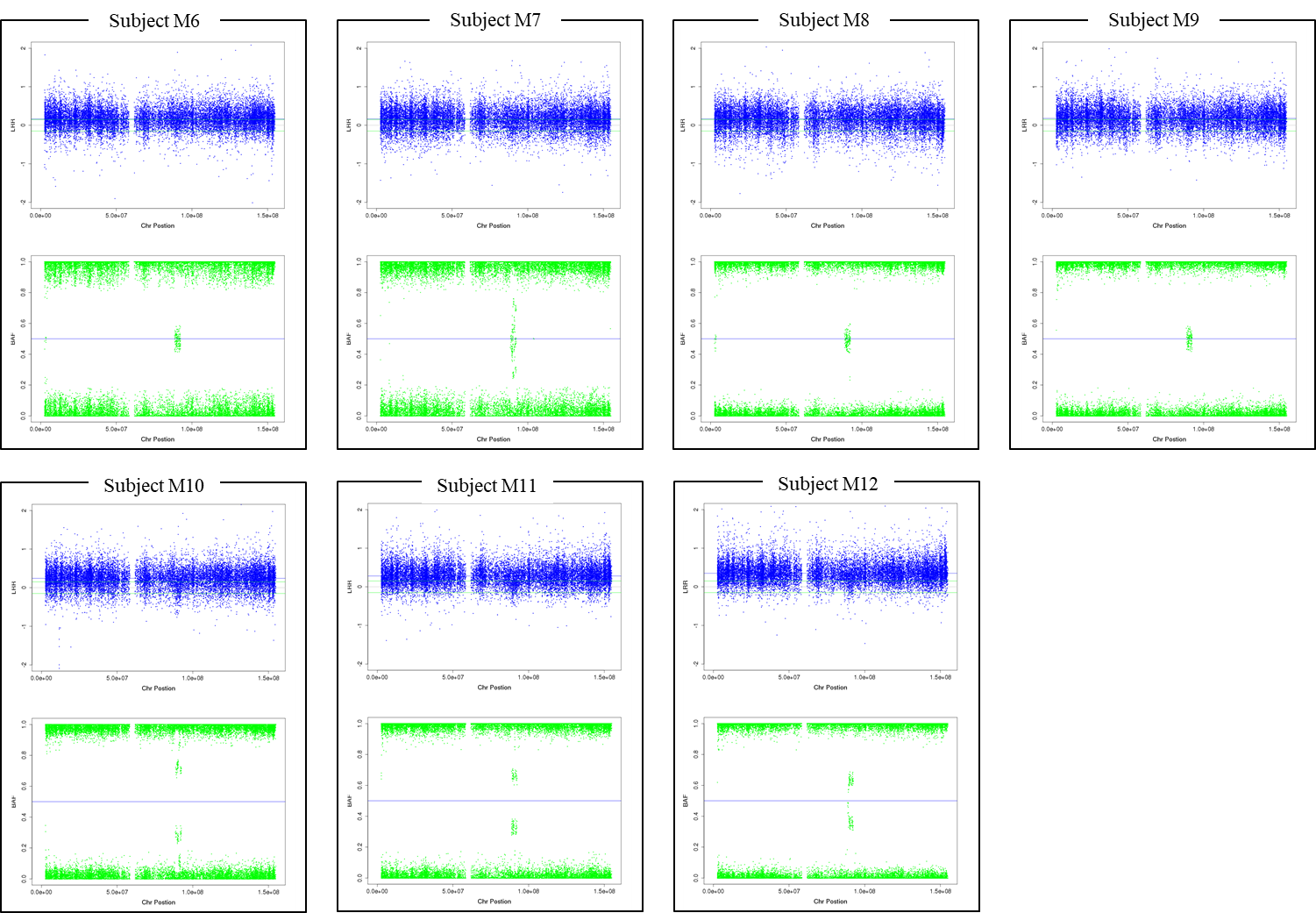


**(B)**

**Supplementary Figure S2.** Chromosome X Log_2_ R Ratio (LRR) and B Allele Frequency (BAF) plots for 24 UK Biobank subjects with constitutional Klinefelter syndrome. The green line represents LRR=0.4 and the blue line represents the median across the X chromosome. This figure continues for the next two pages.


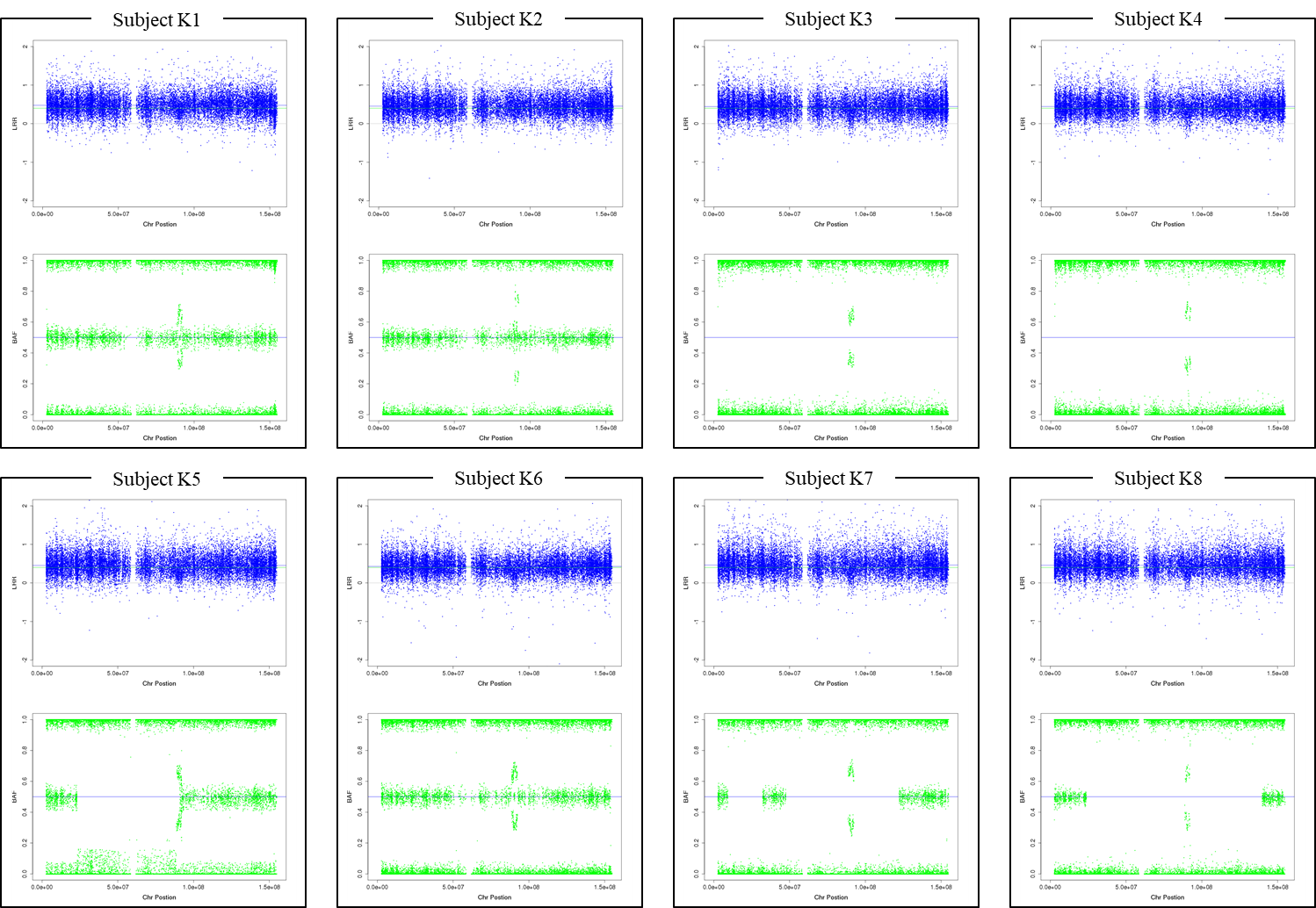


**Supplementary Figure S2 (continued).** Chromosome X Log_2_ R Ratio (LRR) and B Allele Frequency (BAF) plots for 24 UK Biobank subjects with constitutional Klinefelter syndrome. The green line represents LRR=0.4 and the blue line represents the median across the X chromosome. This figure co
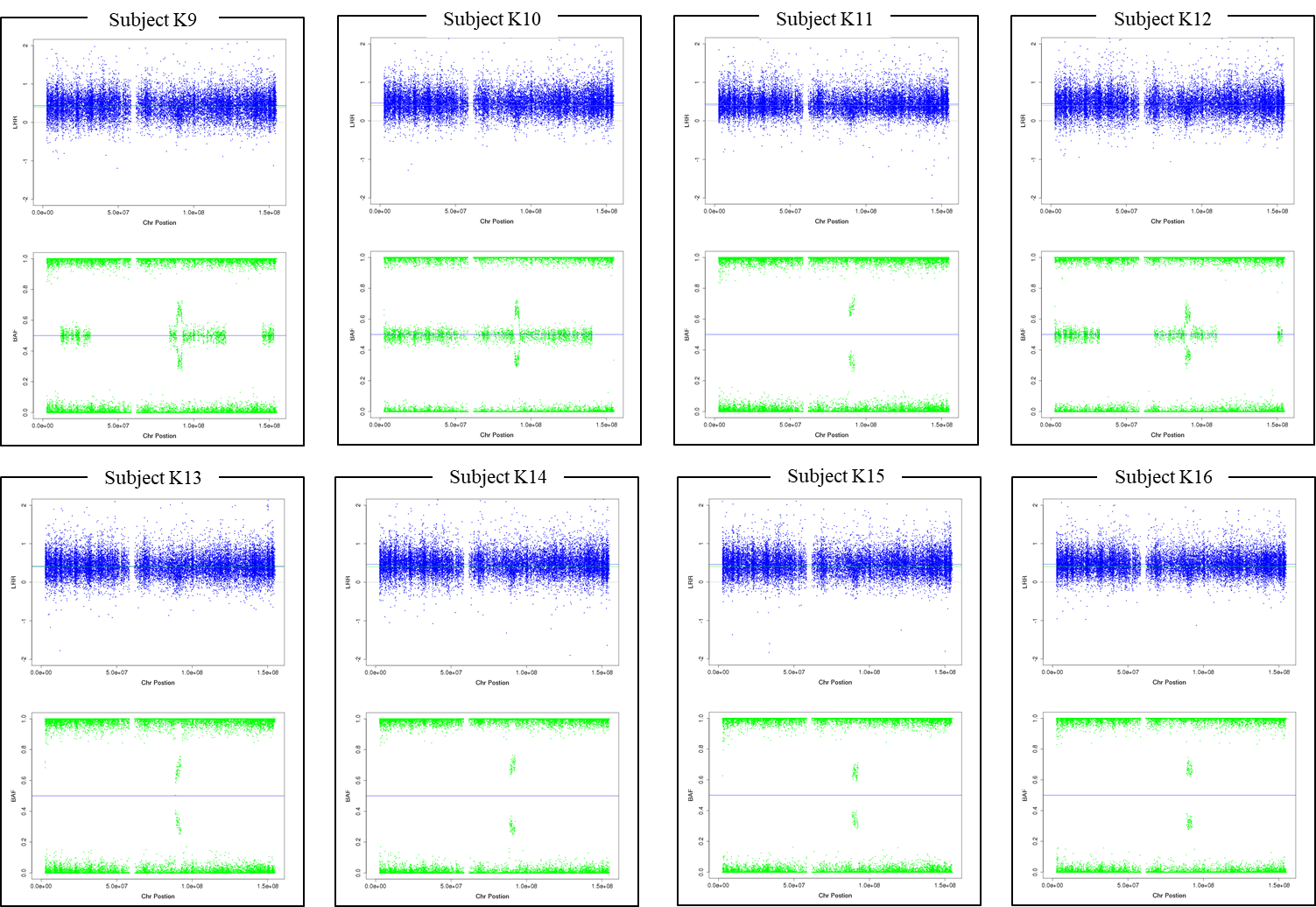
ntinues onto the next page.

**Supplementary Figure S2 (continued).** Chromosome X Log_2_ R Ratio (LRR) and B Allele Frequency (BAF) plots for 24 UK Biobank subjects with constitutional Klinefelter syndrome. The green line represents LRR=0.4 and the blue line represents the median across the X chromosome.


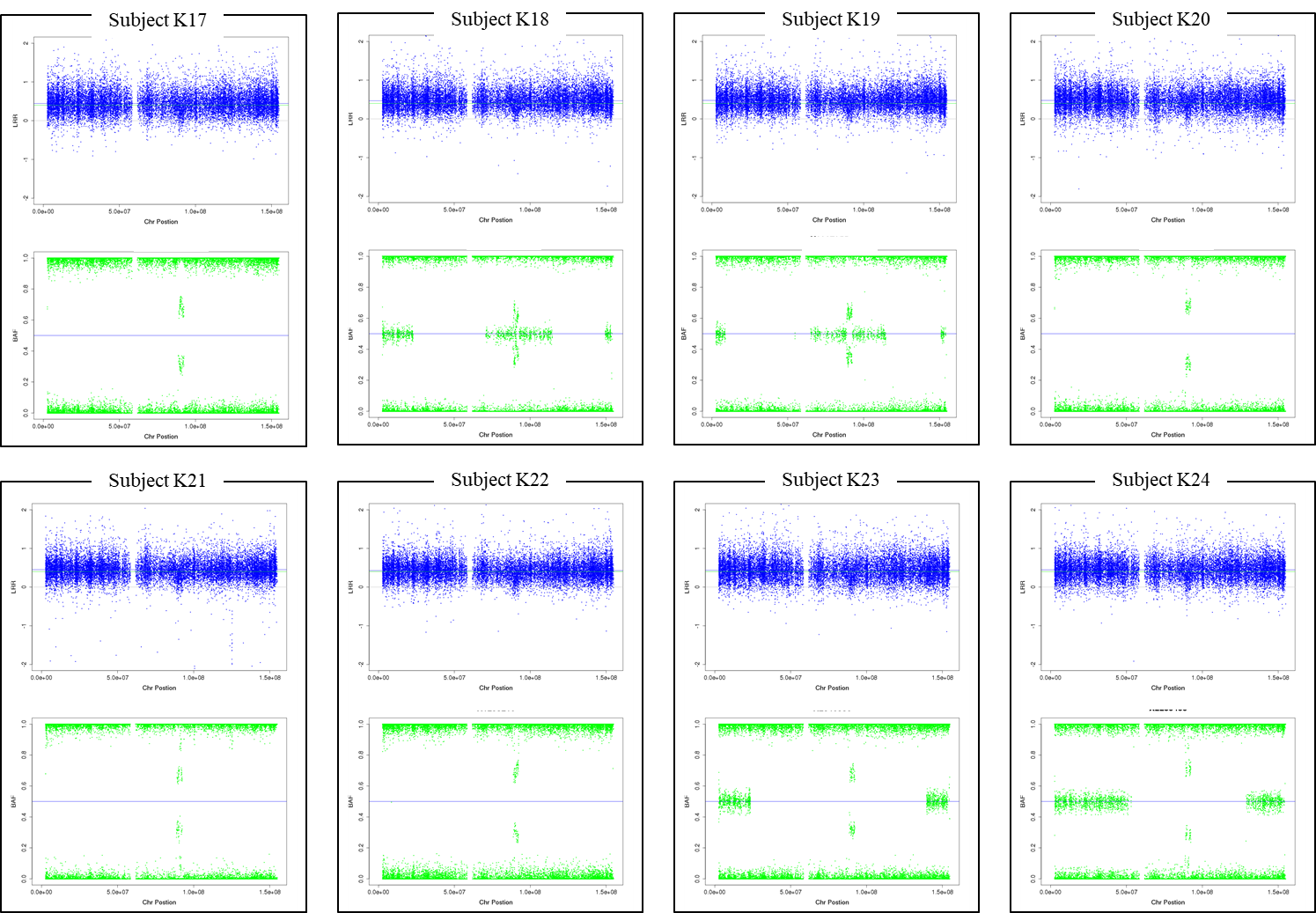


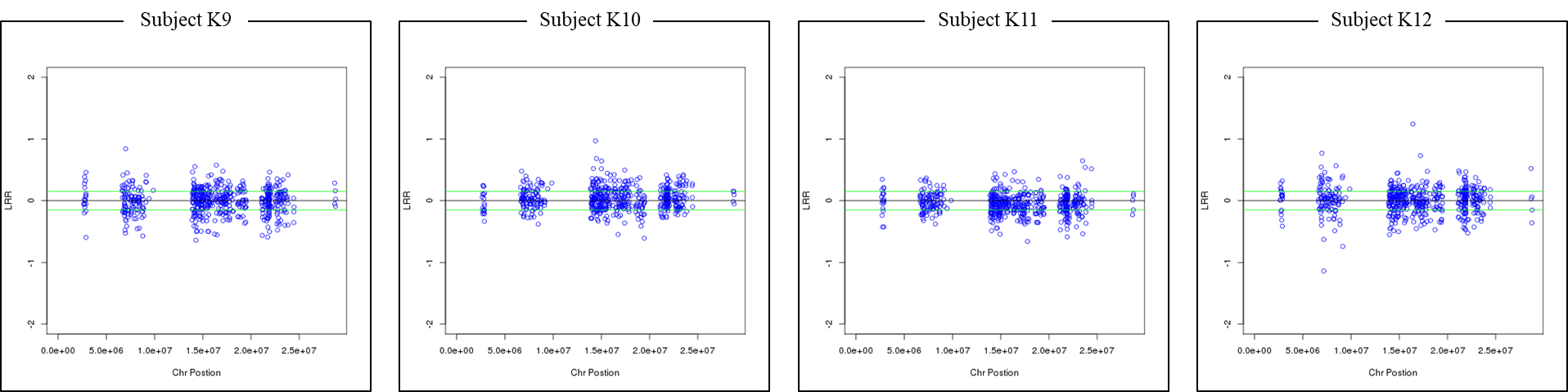

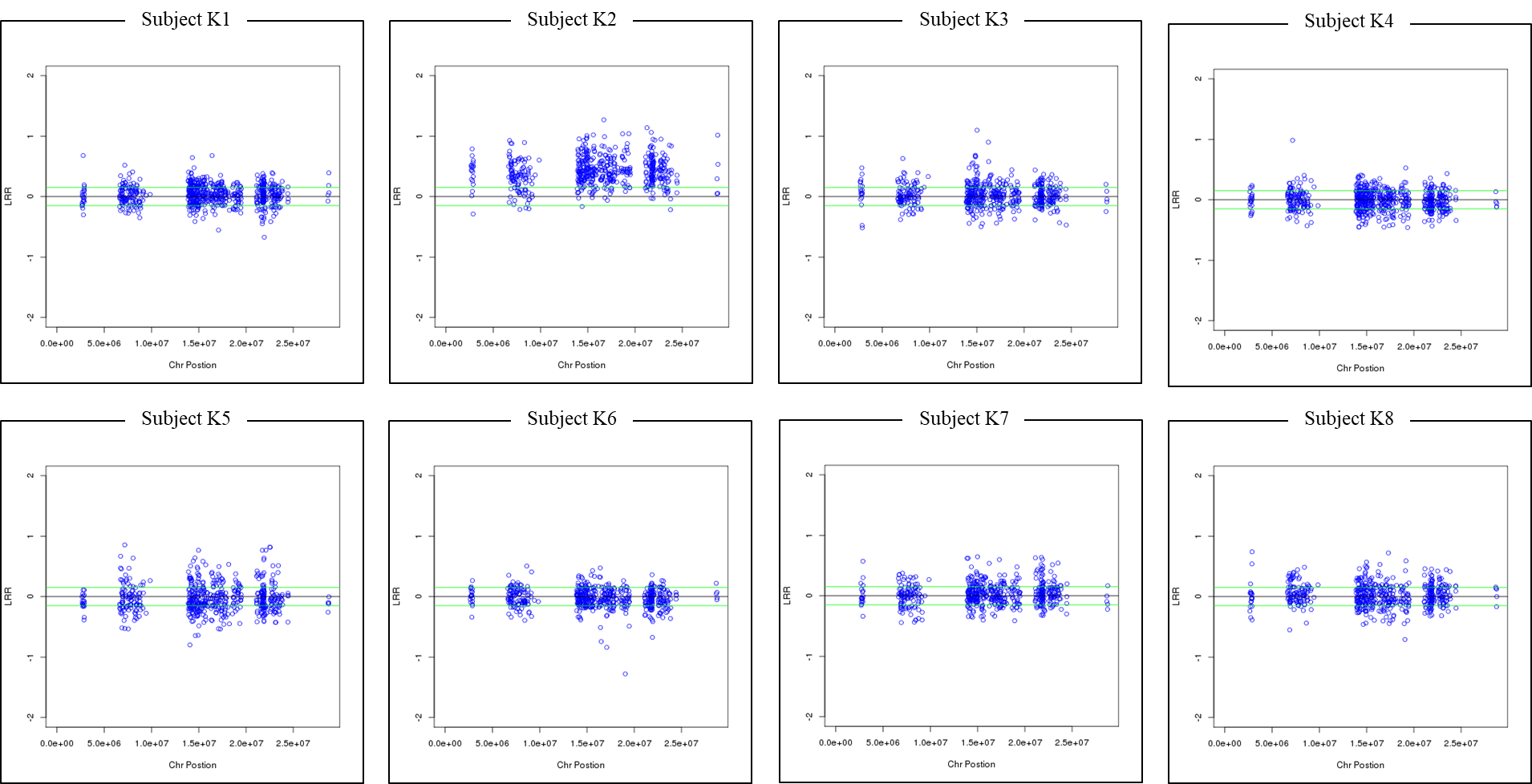
**Supplementary Figure S3**. Chromosome Y Log_2_ R Ratio (LRR) plots for 24 UK Biobank subjects with constitutional Klinefelter syndrome. Green lines indicate an LRR of ±0.15. This figure continues onto the next page.

**Supplementary Figure S3 (continued)**. Chromosome Y Log_2_ R Ratio (LRR) plots for 24 UK Biobank subjects with constitutional Klinefelter syndrome. Green lines indicate an LRR of ±0.15.


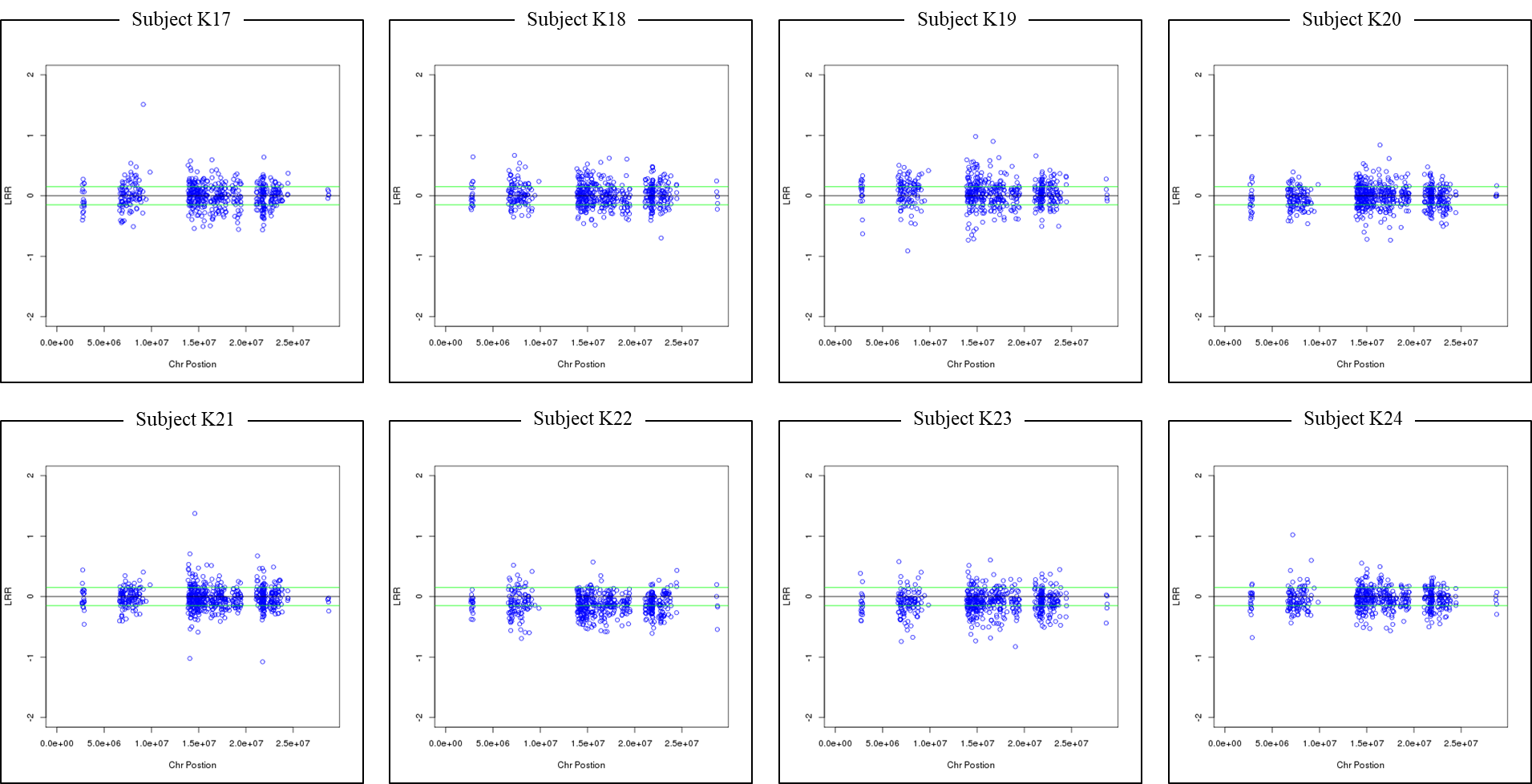

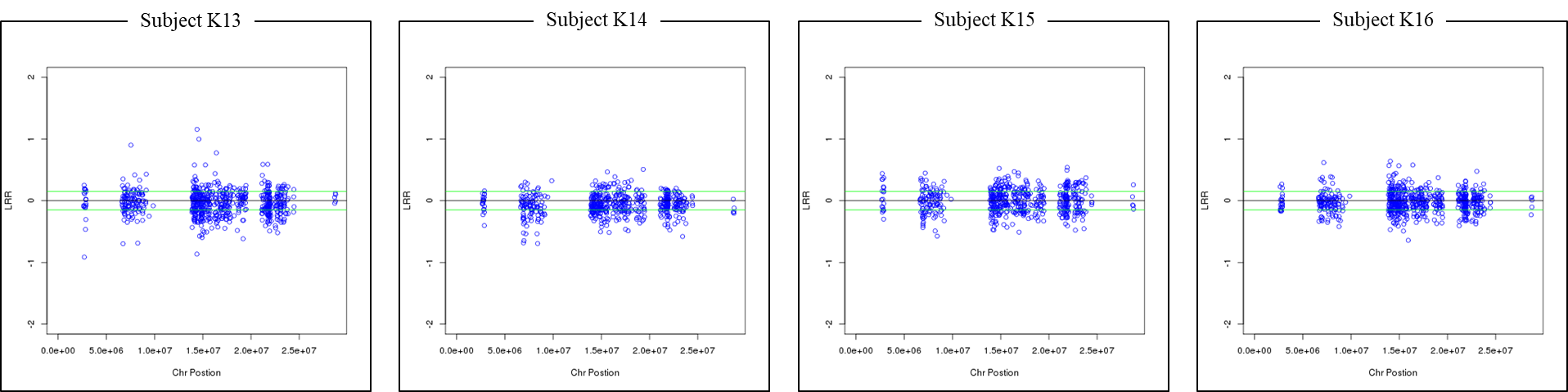


**Supplementary Figure S4**. Raw uncorrected Log_2_ R Ratio (LRR) plots for females (**A**) and males (**B**). Values for mean, median and trim mean (upper and lower 10% of a sample’s LRR data removed) are displayed. Female (diploid) raw LRR values are centered around 0, whereas male (hemizygous) raw LRR values are centered around -0.48. Baseline corrected male LRR values are seen in **Figure 1**. Green lines indicate an LRR of ±0.15 and blue line indicates an LRR of -1.0.

**(A) (B)**


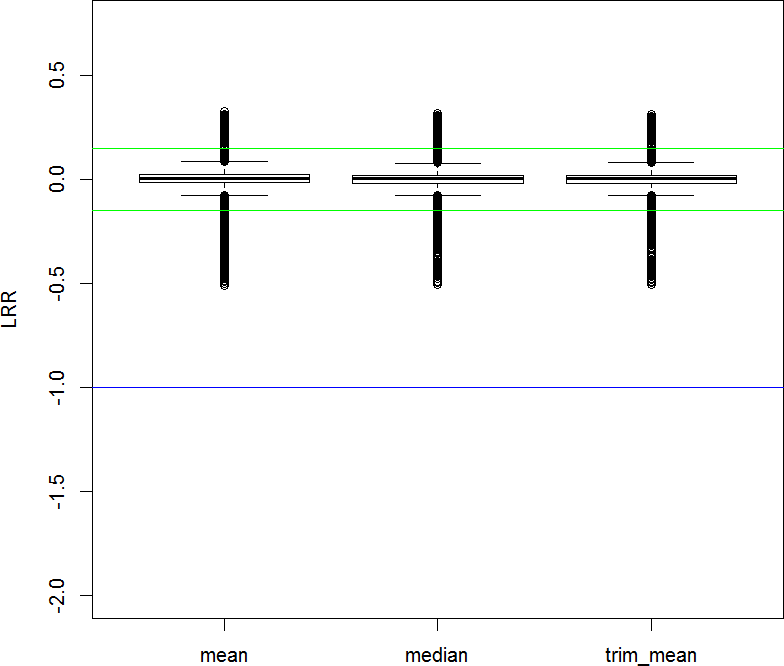

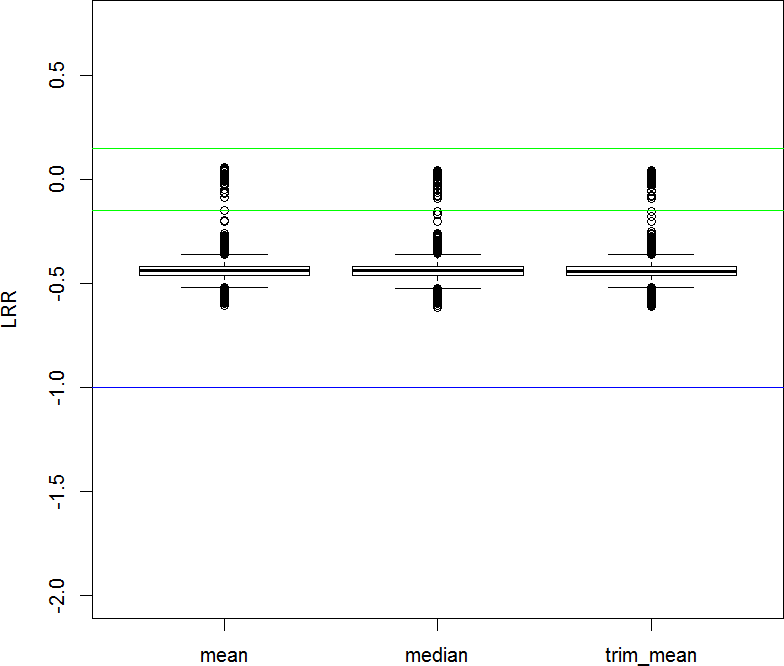

Supplement: Supplementary file 1 — Supplementary Information. [file 41598_2020_80948_MOESM1_ESM.docx]
